# Electrophysiological and Structural Changes in Chinese Patients with LHON

**DOI:** 10.1155/2020/4734276

**Published:** 2020-03-30

**Authors:** Min Wang, Hong Guo, Shiying Li, Gang Wang, Yanling Long, Xiaohong Meng, Bo Liu, Yong Liu, Anthony G. Robson, Zheng Qin Yin

**Affiliations:** ^1^Southwest Hospital/Southwest Eye Hospital, Third Military Medical University (Army Military Medical University), Chongqing, China; ^2^Department of Medical Genetics, Third Military Medical University (Army Military Medical University), Chongqing, China; ^3^Department of Electrophysiology, Moorfields Eye Hospital, London, UK; ^4^Institute of Ophthalmology, University College London, London, UK

## Abstract

**Objective:**

To review retrospectively the electrophysiological and structural changes in 13 Chinese patients with Leber hereditary optic neuropathy (LHON).

**Methods:**

26 eyes of 13 patients with a genetically confirmed diagnosis of LHON were categorized into two groups according to the duration of the disease: group 1 (duration less than 3 months) and group 2 (duration between 3 months and 18 years). Clinical history, comprehensive visual electrophysiology, optical coherence tomography (OCT), and color fundus photography were performed.

**Results:**

Fundoscopy showed optic disc hyperemia in group 1 and optic atrophy in group 2. OCT measures of retinal nerve fiber layer (RNFL) thickness around the optic disc and surrounding macula were normal in group 1 but reduced in group 2 (10 of 10 eyes). The thickness of the retinal ganglion cell layer (GCL) plus inner plexiform layer (IPL) surrounding the macula reduced significantly in group 1 and group 2 compared with a healthy control group. Pattern ERG (PERG) P50 amplitude was normal, but the N95/P50 ratio reduced in most of group 1 (4 of 5 eyes) and in all of group 2 (11 eyes). PERG P50 peak time was abnormally short in group 2. Multifocal electroretinography (mfERG) showed subnormal responses associated with ring 1 (the central area) and ring 2 in group 1 and reductions in rings 1, 2, and 3 in group 2.

**Conclusion:**

The study highlights differences in retinal structure and function between the acute and chronic stages of LHON in a group of Chinese patients. There is PERG evidence of retinal ganglion cell dysfunction and OCT evidence of GCL + IPL thinning in both groups, but there is additional peripapillary RNFL loss in the chronic stage, associated with more severe RGC dysfunction. There is multifocal ERG evidence of localized macular dysfunction in both acute and chronic groups. The study highlights the importance of comprehensive electrophysiological and structural assessments of the retina in LHON and is pertinent to studies that aim to monitor disease progression or the effects of future therapeutic interventions.

## 1. Introduction

Leber hereditary optic neuropathy (LHON) was first described by Theodor Leber in 1871 [1] and is one of the most common disorders caused by mitochondrial DNA (mtDNA) mutation. Over 95% of LHON pedigrees harbor one of three mtDNA point mutations (G3460A, G11778A, and T14484C), which all involve genes encoding complex I subunits of the mitochondrial respiratory chain [2]. The retinal ganglion cell (RGC) system is particularly vulnerable to mitochondrial dysfunction, and optic atrophy is a frequent characteristic of mitochondrial and neurodegenerative diseases [3, 4]. An intriguing feature of LHON is that approximately only 50% of males and 10% of females who harbor a pathogenic mtDNA mutation develop optic neuropathy. Pathogenic mtDNA mutations are necessary for the development of LHON, but other factors must be responsible for the variable penetrance and the male predominance of the condition. Studies have examined the possible influence of an *X*-linked visual loss susceptibility locus, impaired mitochondrial respiratory chain activity, mtDNA heteroplasmy, environmental factors, and autoimmune factors [5, 6]. The clinical characteristics of LHON are well documented [7–10], but fewer studies have compared visual electrophysiology with optical coherence tomography (OCT) of the retinal ganglion cell layer (GCL) and retinal nerve fiber layer (RNFL) in different stages of the disease. The aim of this study is to compare the retinal structure and function in both the acute and chronic stages of LHON in Chinese patients. The influence of the disease stage is examined by detailed OCT measures of macular and peripapillary retinal structure and by comprehensive electrophysiology, including pattern and multifocal electroretinography (PERG; mfERG).

## 2. Materials and Methods

Thirteen Chinese patients with LHON were ascertained in the Department of Ophthalmology at the Southwest Hospital in Chongqing between April 2013 and April 2015. Informed consent was obtained from all patients. Each subject was treated in accordance with the tenets of the Declaration of Helsinki and the study was approved in advance by the Ethics Committee of Third Military Medical University, Chongqing, China.

There were 11 male and 2 female patients with a median age of 18 years (range 8–43 years). Patients were divided into two groups according to the disease duration: group 1 (acute stage; duration less than 3 months) and group 2 (chronic stage; duration 3 months to 18 years). The duration was measured from and the date of onset of visual symptoms, as reported by the patients at presentation. Group 1 included 7 eyes and group 2 included 19 eyes. One patient examined in the acute stage was monitored for 3 years. All patients were screened for the 7 primary and 9 secondary mtDNA mutations, most commonly involved in LHON as reported in ITOMAP (http://www.mitomap.org).

Subjects underwent a clinical and ophthalmological examination including visual acuity assessment, fundoscopy, fundus fluorescein angiography (FFA), macular, and peripapillary OCT. The OCT (Topcon3D-1000, Topcon Corporation, Japan) was performed on the macula (6 mm × 6 mm; centered on the fovea) and a macular thickness map used to measure the thickness of the retinal ganglion cell layer plus inner plexiform layer (GCL + ICL) and the thickness of the retinal nerve fiber layer (RNFL). Scanning of the peripapillary area was used to assess the RNFL layer surrounding the optic disc (RNFL-OCT).

Electrophysiological investigations included full-field electroretinography (FERG), PERG, and visual evoked potential (VEP) testing (Espion system, Diagnosys LLC, Lowell, MA, USA). Multifocal ERG testing was additionally performed using a 103-element scaled stimulus array. The mfERG system incorporated a real-time fundus camera, allowing fixation-dependent stimulus placement to be monitored during the examination (Veris system, Electro-Diagnostic Imaging, Inc., Burlingame, CA, USA; CCD Camera, Hitachi Kokusai Electric Inc, Japan). All tests were performed according to the International Society for Clinical Electrophysiology of Vision (ISCEV) Standards [11–14], with the exception of the PERG, performed using a large stimulus field size (24 degrees × 18 degrees). Recordings were compared with normal laboratory-specific controls. Differences between patients and age-matched controls were tested statistically using an independent two-sample *t*-test performed in SPSS Statistics software (Version 19, IBM, Armonk, NY, USA).

## 3. Results

Genetic testing revealed mtDNA mutation 11778 in seven patients, 3460 in two, 3497 in two, and mutations 3635 and 3316 in one, and mutation 3394 in one patient.

**Figure 1:**
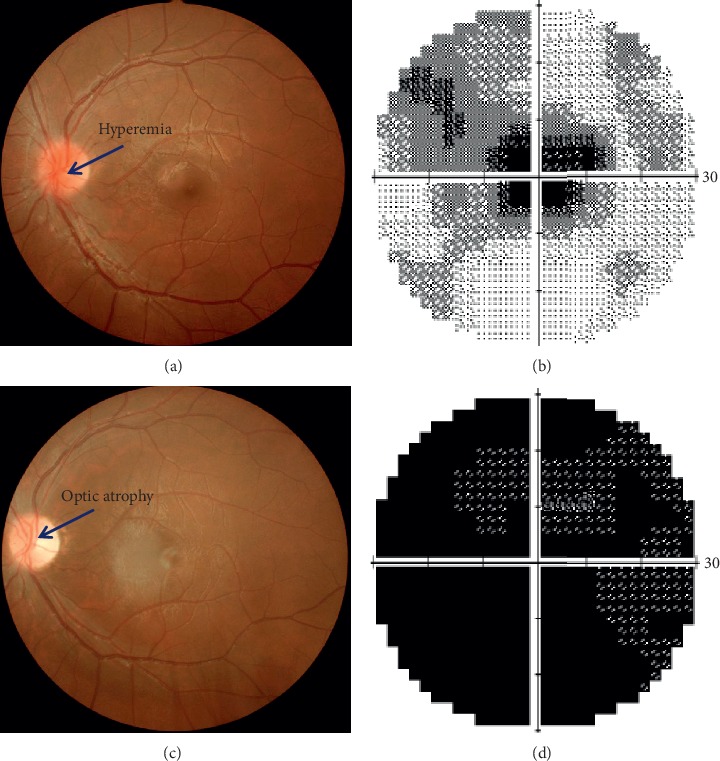
Color fundus photographs and corresponding visual fields obtained from patient 1 listed in Table 1. (a) Fundus photo, (b) visual field at acute stage (1 month after onset), (c) fundus photo, and (d) visual field at chronic stage (5 months after onset). Left eye, 16 years old, BCVA: 6/120 (acute stage); 6/300 (chronic stage).

The clinical and electrophysiological characteristics of the patients are summarized in Table 1. Visual acuity was severely impaired in all patients. On fundoscopy, there was optic disc hyperemia in Group 1 (all 7 eyes) and disc pallor in Group 2 (all 19 eyes; see Figure 1 for examples). Visual field testing showed central defects and blind spot enlargement in all patients.

**Figure 2:**
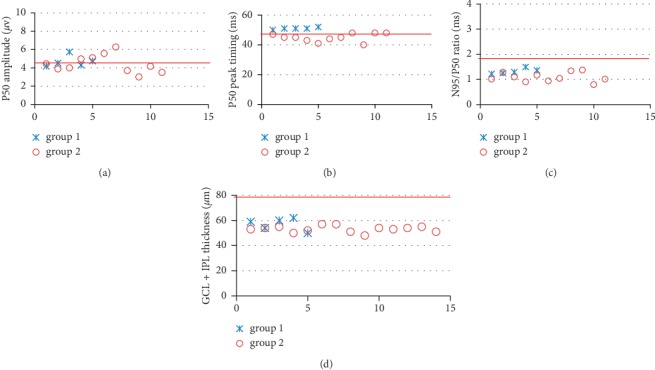
(a) Pattern ERG P50 amplitude, (b) P50 peak timing, (c) N95/P50 ratio, and (d) the GCL + IPL thickness surrounding macula of all the eyes that underwent testing were plotted. The N95/P50 ratio reduced significantly in group 1 and group 2 compared with normal (*p* < 0.001 for each group) and more severely reduced in most group 2, consistent with thinning of GCL + IPL thickness surrounding macula. Individuals from each group are numbered (abscissa) and plotted as crosses (acute group/group 1) and open circles (chronic group/group 2). Red line in each picture means average normal value. And the normal range is average normal value ± SD of each index.

**Figure 3:**
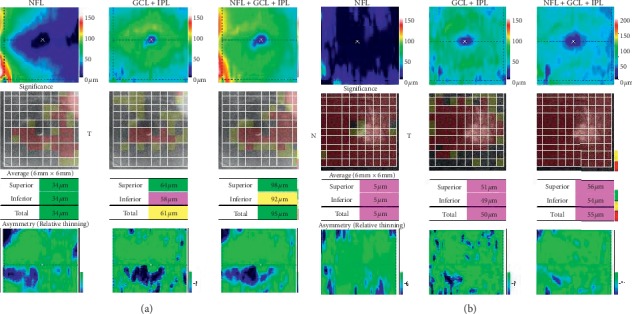
Macular thickness OCT obtained from patient 1 in Table 1. (a) Acute stage, no reduced RNFL layer thickness around macula, reduced GCL + IPL layer thickness. (b) Chronic stage, reduced RNFL layer thickness around macula, and GCL + IPL layer thickness. Left eye, 16 years old, BCVA: 6/120 (acute stage); 6/300 (chronic stage).

The OCT data are summarized in Table 2. The RNFL thickness around the optic disc was normal in group 1 and abnormal in group 2. RNFL thickness surrounding the macula was normal in group 1 compared to controls but reduced significantly in group 2. The thickness of the GCL + IPL layer surrounding macula was reduced in both groups compared with normal (Table 2; Figure 2). An example of macular OCT, performed in the acute and chronic stages in a single individual, is shown in Figure 3.

**Figure 4:**
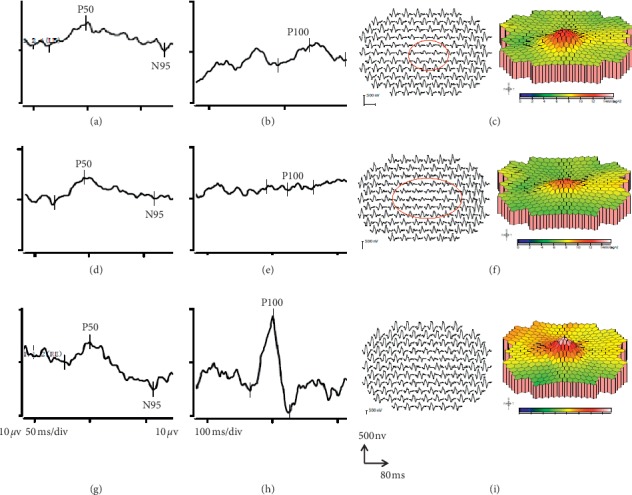
Electrophysiological recordings obtained from patient 1 in Table 1. (a)PERG, (b)PVEP, (c) mfERG at acute stage, (d) PERG, (e) PVEP, (f) mfERG at chronic stage, (g) PERG, (h) PVEP, and (i) mfERG of normal control eye. The N95 component amplitude reduced both in the acute and chronic stage. The amplitude density of inner rings of the MFERG reduced both in the acute and chronic stages. BCVA: 6/120 (acute stage); 6/300 (chronic stage). PERG recordings were reproducible.

Pattern ERG P50 amplitude was normal in both groups, but the N95/P50 ratio was reduced significantly in group 1 and group 2 compared with the (normative) control group (Figure 2; Table 3; *p* < 0.001 for each group). The N95/P50 ratio was more severely reduced in most group 2 compared with group 1 cases. The P50 peak time was normal in group 1 but was abnormally shortened in most eyes in group 2 with a mean difference from the normal mean of 4.7 ms (Figure 2; Table 3; acute vs control, *p*=0.141; chronic vs control, *p*=0.001). An example of pattern ERG data recorded from one individual is shown in Figure 4.

**Figure 5:**
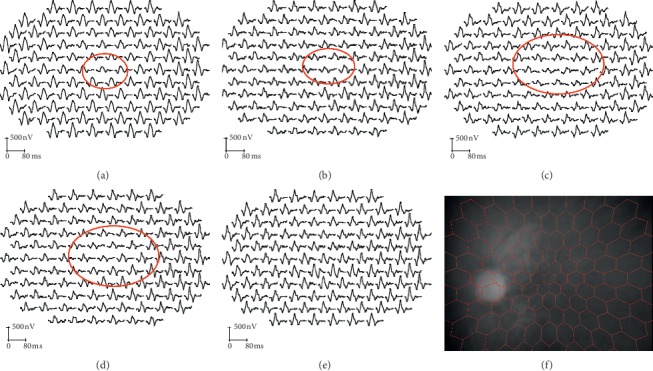
Examples of trace array in mfERG in both acute and chronic stage. (a, b) Two eyes at acute stage, (c, d) two eyes at chronic stage, (e) normal eye, and (f) eye fundus monitoring with camera when recording the traces. As shown above, amplitude density reduced in the inner rings in both the acute and chronic stages.

Examples of mfERGs recorded in groups 1 and 2 are shown in Figures 4 and 5 and all mfERG data are summarized in Table 4. The amplitude density of the central (ring 1 and 2) multifocal ERG in group 1 was significantly subnormal in 4 and 3 of 5 eyes compared with the control data, respectively (Table 4; *p* < 0.01, *p* < 0.05); responses in Ring 3 to Ring 6 were not significantly reduced (*p* > 0.01). In Group 2, the amplitude density in Rings 1, 2, and 3 was significantly reduced in 16, 15, and 13 of 17 eyes, respectively (Table 4; *p* < 0.001); responses in Rings 4 to 6 were not significantly different from normal (*p* > 0.01). The mfERG reductions in Rings 1 and 2 were significantly more severe in group 2 compared with group 1 (*p* < 0.05). Structural and functional changes are summarized in Table 5, including measurements of RNFL and GCL + IPL thickness in the macular OCT, PERG P50, and N95/P50 ratio changes, and mfERG amplitude reductions.

The amplitude of the pattern reversal VEP P100 component was decreased and the peak time delayed by a mean of 19.5 ms in 5/5 eyes in group 1; responses were undetectable in 3/8 eyes in group 2, and in the other 5 eyes the amplitude was decreased and the peak times delayed by a mean of 21.1 ms. The ISCEV-standard full-field ERG components were normal in all patients in both groups. There was no significant correlation between the severity of VEP, PERG, and mfERG abnormalities.

## 4. Discussion

This study compares cases of genetically confirmed LHON in acute and chronic stages according to detailed OCT scans of the macula and peripapillary regions and according to comprehensive electrophysiological measures of retinal function, including the pattern ERG, full-field ERG, and multifocal ERG. Both acute and chronic groups show OCT evidence of inner retinal loss and both show PERG N95 reduction consistent with RGC dysfunction. In chronic cases, there is additional macular and peripapillary RNFL loss associated with more severe RGC dysfunction in most, manifest as PERG N95 reduction and shortening of P50 timing. A novel finding is that 20 of 21 cases show mfERG evidence of additional localized central macular dysfunction, more severe and extensive in chronic than in acute cases.

LHON is one of the most common primary mitochondrial DNA (mtDNA) disorders. Retinal ganglion cells (RGCs) are exquisitely sensitive to mitochondrial dysfunction, and the papillomacular bundle is affected early and severely in LHON. The preferential involvement of the RGCs within the papillomacular bundle is likely related to the relatively small calibre of the axons and limited mitochondrial energy reserves [15]. Two major mechanisms have been proposed to precipitate RGC loss in LHON: a biochemical respiratory chain defect and increased levels of reactive oxygen species (ROS) [16]. Not all LHON mutation carriers will experience visual loss during their lifetime. Three mtDNA point mutations (G3460A, G11778A, and T14484C), which all involve complex I subunits of the mitochondrial respiratory chain, account for the vast majority of LHON cases, as reported in MITOMAP (http://www.mitomap.org). Rarer mtDNA mutations have also been confirmed as causing the LHON phenotype, having been reported in more than one pedigree, and showing clear segregation with affected disease status. In our study, 9 patients carried G3460A or G11778A mutation but none were identified that carried the T14484C mutation, possibly due to the small sample size. Other studies have identified Chinese patients with LHON that harbor the T14484C mutation [17].

Fundoscopy showed optic disc hyperemia in the acute stage (≤3 months from onset) in patients with LHON and optic atrophy in the chronic stage (>3 months from onset). Central field defects and blind spot enlargement were common findings, in keeping with previous investigations [18]. The current study showed that that the thickness of the RNFL around the optic disc and surrounding the macula did not reduce in the acute stage but reduced significantly in the chronic stage of the disease, corroborating previous reports [19, 20].

The pattern ERG N95 and approximately 70% of P50 originate in the retinal ganglion cells. There was a marked reduction in the PERG N95/P50 ratio in both acute and chronic LHON groups, indicating RGC dysfunction and consistent with thinning of GCL + IPL layer. PERG N95 loss can occur in other forms of optic neuropathy but usually in longstanding disease (minimum 4–6 weeks but often longer) due to retrograde degeneration of the RGCs; N95 reduction in the acute stages for all patients is in keeping with primary ganglion cell disease. Additional shortening of P50 was a consistent feature in those with a history of LHON longer than 3 months, likely representing an additional loss of the RGC contribution to P50 and consistent with relatively rapid progression and worsening involvement of the RGCs.

A novel finding was that mfERG showed a reduction over central areas in both acute and chronic cases. Multifocal ERGs can be influenced by poor fixation, particularly if vision is impaired, but in the current study the mfERG system incorporated a fundus camera, allowing fixation-dependent stimulus placement on the fundus to be monitored during the examination. The mfERG scalar plots and relative preservation of PERG P50 (Figure 4) are consistent with adequate central fixation. The mfERG N1 and P1 components arise largely in the On and Off bipolar cells with a cone photoreceptor contribution to N1 [21]. The abnormal mfERGs suggest dysfunction of either the macular cones or macular bipolar cells in the absence of fundus change (an occult maculopathy) or a previously unsuspected contribution of the RGCs to the first-order mfERG. It is notable that macular dysfunction was not severe enough to significantly attenuate the PERG P50, presumably due to lower spatial resolution of the large-field PERG technique. An important implication is that macular dysfunction may contribute to visual acuity reduction and may influence other psychophysical tests of central macular function and pattern VEP findings. The latter tests are often used in the assessment of optic neuropathy and RGC dysfunction and may be used to monitor disease progression or treatment efficacy. The severity of pattern VEP abnormalities in the current study may largely be due to optic nerve dysfunction but may also partly be attributable to occult maculopathy. In LHON, RGC dysfunction may be of primary importance but it would seem prudent to additionally monitor macular cone and bipolar cell function objectively, to better understand and more accurately monitor the pathophysiology of disease and potential efficacy of future treatments.

The study highlights differences in retinal structure and function between the acute and chronic stages that are pertinent to investigations that aim to monitor disease severity and progression or efficacy of future treatments. The visual prognosis in LHON is poor and most patients remain legally blind. Owing to the limited understanding of the mechanisms of LHON, treatment options are still in their infancy. Supportive measures, such as low-vision aids and recognition and therapy of associated treatable systemic abnormalities, remain the primary focus for the clinician. Idebenone (a quinone analog of coenzyme Q10) has been reported to be effective in increasing the rate of recovery in LHON especially in patients with discordant visual acuity and if the patients are treated early in the disease course [22] As a better understanding of the pathophysiology is gained, novel, more-effective therapies are likely to emerge, and more targeted treatments developed. The ongoing development of animal models for the hereditary optic neuropathies will facilitate the translation from bench to bedside. Gene therapy is particularly encouraging for those patients whose phenotypic expression is limited to the optic nerve and retinal ganglion cells, as the affected neurons are anatomically accessible to directed therapy [23–27]. The value of the comprehensive electrophysiological assessment is highlighted, for objectively characterizing the levels and severity of dysfunction within the retina, and for the objective monitoring of efficacy in future therapeutic interventions.

## Figures and Tables

**Table 1:** Summary of genetic, functional, and structural findings in patients with LHON.

	Patient number	Gender	Eye	Age	Gene mutation	Snellen BCVA	PVEP	PERG			mfERG	OCT		
							Peak time delayed (ms)^*∗*^	P50 amplitude reduced	N95/P50 amplitude reduced	Peak time shortening	Ring 1-2 amplitude decreased	GCL + IPL thickness thinning in macula	RNFL thickness thinning in macula	RNFL around optic disc
Acute	1	M	L	16	11778	6/120	35	−	+	−	+	+	+	−
Chronic	1	M	R	16	11778	6/300		−	+	+	+	+	+	+
Acute	2	M	R	14	11778	6/150	20	−	+	−	+	+	−	
Acute	2	M	L	14	11778	6/40	16	−	−	−	+	+	−	
Acute	3	M	R	13	3635, 3316	6/150	12	−	+	−	+	+	−	−
Acute	3	M	L	13	3635, 3316	6/200	12	−	+	−	−	+	+	−
Acute	4	M	R	38	3497	CF								
Acute	4	M	L	38	3497	6/600								
Chronic	5	M	R	19	11778	6/200		−	+	+	+	+	+	+
Chronic	5	M	L	19	11778	6/200		−	+	+	+	+	+	+
Chronic	6	F	R	18	3460	6/40	18	−	+	+	+	+	+	
Chronic	6	F	L	18	3460	6/60	19	−	+	+	+	+	+	
Chronic	7	M	R	15	11778	CF	27	−	+	+	+	+	+	+
Chronic	7	M	L	15	11778	6/600	25	−	+	+	+	+	+	+
Chronic	8	F	L	43	3460	6/20					+			
Chronic	8	F	R	43	3460	6/120					+			
Chronic	9	M	L	38	11778	6/300		−	+	+	+	+	+	+
Chronic	9	M	R	38	11778	CF		+	+	+	+	+	+	+
Chronic	10	M	L	8	3394	6/600					+	+	+	+
Chronic	10	M	R	8	3394	6/60					+	+	+	+
Chronic	11	M	L	29	3497	6/9					+	+	+	+
Chronic	11	M	R	29	3497	6/60						+	+	+
Chronic	12	M	L	18	11778	6/60		−	+	+	+	+	+	+
Chronic	12	M	R	18	11778	CF		−	+	+	+	+	+	+
Chronic	13	M	L	41	11778	6/600								
Chronic	13	M	R	41	11778	6/60								

Notes: “−” signifies normal findings; “+” signifies abnormal findings; absence of data indicates that tests were not performed; “CF” indicates “counting fingers visual acuity”; ^*∗*^PVEP peak time delay indicates the time above the upper limit of normal in the age-matched control group.

**Table 2:** Optical coherence tomography (OCT) measures of RNFL thickness around the optic disc (GCL + IPL) and RNFL thickness surrounding macula in group 1 (acute cases), group 2 (chronic cases), and in the control group.

		Group 1	Group 2	Control group
RNFL around optic disc	Number of eyes	3	13	10
	RNFL thickness (*μ*m)	117 ± 12.1	66.1 ± 16.2^*∗*^	105.7 ± 8.1

Macula thickness	Number of eyes	5	15	10
	GCL + IPL thickness (*μ*m)	57.0 ± 4.9^*∗*^	53.1 ± 2.5^*∗*^	71.7 ± 3.9
	RNFL thickness (*μ*m)	31.8 ± 9.5	7.2 ± 7.1^*∗*^	35.4 ± 3.9

RNFL thickness around optic disc did not reduce in group 1 but reduced in group 2 (Group 1 vs. control, *p*=0.08; group 2 vs. control, *p* < 0.001^*∗*^). The GCL + IPL thickness surrounding macular area (6 mm × 6 mm, centered on fovea) reduced in both groups (Group 1 vs. control, *p* < 0.001^*∗*^; group 2 vs. control, *p* < 0.001^*∗*^). The RNFL thickness surrounding macular area did not reduce in group 1 (*p*=0.454), but reduced in group 2 (*p* < 0.001^*∗*^). RNFL: retinal nerve fiber layer; GCL + IPL: ganglion cell layer plus inner plexiform layer.

**Table 3:** PERG P50 amplitude, N95/P50 ratio, and P50 peak time in group 1 and group 2 compared with the normal group.

	Eyes	Group 1	Group 2	Control group
		5	11	15
PERG	P50 amplitude (*μ*v)	4.6 ± 0.7	4.5 ± 0.9	5.2 ± 1.1
	N95/P50 ratio	1.32 ± 0.11^*∗*^	1.12 ± 0.16^*∗*^	1.74 ± 0.17
	P50 peak time (ms)	51.0 ± 0.7	44.9 ± 2.8^*∗*^	49.6 ± 3.3

The PERG P50 component amplitude was normal compared with controls in both groups (group 1 vs control, *p*=0.278; group 2 vs control *p*=0.128). The PERG N95/P50 ratio was significantly decreased in group 1 and group 2 compared with control group (*p* < 0.001^*∗*^ for each group). P50 peak time was normal in group 1 and abnormally shortened in group 2 (group 1 vs control, *p*=0.141; group 2 vs control *p*=0.001^*∗*^).

**Table 4:** Amplitude density of mfERG for 6 rings.

		Group 1	Group 2	Control group
Amplitude density of MfERG (*μ*v/deg)	Ring 1	30.7 ± 8.5^*∗*^	20.1 ± 8.8^*∗*^	42.2 ± 6.0
	Ring 2	25.3 ± 7.6^*∗*^	18.0 ± 6.7^*∗*^	31.9 ± 3.2
	Ring 3	20.6 ± 6.1	16.8 ± 6.0^*∗*^	23.9 ± 1.8
	Ring 4	16.9 ± 4.9	15.1 ± 5.5	19.1 ± 1.6
	Ring 5	14.0 ± 4.4	14.2 ± 5.3	16.1 ± 1.8
	Ring 6	12.9 ± 4.4	13.7 ± 6.3	14.3 ± 2.5

Number of eyes		5	17	10

In group 1, the mean amplitude density of rings 1 and 2 were reduced significantly compared with controls (*p*=0.009^*∗*^, *p*=0.032^*∗*^ respectively). In group 2, the mean amplitude density of rings 1, 2, and 3 were reduced significantly compared with controls (*p* < 0.001, *p* < 0.001, and*p* < 0.001^*∗*^, respectively).

**Table 5:** Summary of structural and functional characteristics.

Group 1	Group 2
*Structural*	*Structural*
OCT RNFL normal (3 of 5 eyes)	OCT RNFL reduced (15 of 15 eyes)
OCT GCL + IPL reduced (5 of 5 eyes)	OCT GCL + IPL reduced (15 of 15 eyes)

*Functional*	*Functional*
P50 normal (5 of 5 eyes)	P50 normal (10 of 11 eyes)
N95/P50 reduced (4 of 5 eyes)	N95/P50 reduced (11 of 11 eyes)
AD of ring 1 reduced (4 of 5 eyes)	AD of ring 1 reduced (16 of 17 eyes)
AD of ring 2 reduced (3 of 5 eyes)	AD of ring 1 reduced (15 of 17 eyes)
AD of ring 3 reduced (3 of 5 eyes)	AD of ring 1 reduced (13 of 17 eyes)

Structural measures are shown for “OCT RNFL” (thickness of the RNFL in macular OCT) and “OCT GCL + IPL” (thickness of GCL + IPL layer in macular OCT). Functional measures include the pattern ERG P50 amplitude, N95 : P50 amplitude ratio, and amplitude density (AD) of the mfERG in ring 1 (fovea) and rings 2 and 3.

## Data Availability

The clinical data used to support the findings of this study are available from the corresponding author upon request.
